# Genetic characterization of dilated cardiomyopathy patients undergoing heart transplantation in the Chinese population by whole-exome sequencing

**DOI:** 10.1186/s12967-023-04282-5

**Published:** 2023-07-17

**Authors:** Hong Lian, Shen Song, Wenzheng Chen, Anteng Shi, Haobin Jiang, Shengshou Hu

**Affiliations:** 1 State Key Laboratory of Cardiovascular Disease, Fuwai Hospital, National Center for Cardiovascular Disease, Chinese Academy of Medical Sciences and Peking Union Medical College, Beijing, 100037 China; 2 Beijing Key Laboratory of Preclinical Research and Evaluation for Cardiovascular Implant Materials, Animal Experimental Center, Fuwai Hospital, National Center for Cardiovascular Disease, Chinese Academy of Medical Sciences and Peking Union Medical College, Beijing, 100037 China

**Keywords:** Dilated cardiomyopathy, Heart transplantation, Whole-exome sequencing

## Abstract

**Background:**

Dilated cardiomyopathy (DCM) is one of the most frequent causes of heart failure and heart transplantation (HTx). The genetic basis of DCM among patients undergoing HTx remains to be further studied. This study aimed to characterize the genetic basis of DCM HTx in the Chinese population.

**Methods:**

In total, 208 unrelated DCM patients who underwent HTx at Fuwai Hospital between June 2004 and June 2017 were included in this study. Whole-exome sequencing (WES) was performed for all patients. Gene burden analysis, variant classification, and genotype–phenotype correlation analysis were subsequently performed.

**Results:**

After completing the bioinformatics analysis, gene burden analysis suggested that titin (*TTN*), filamin C (*FLNC*) and lamin A/C (*LMNA*) were significantly enriched with rare protein-altering variants. The frequencies of *TTN* and *FLNC* truncating variants in our cohort were 18.8% and 8.7%, respectively. Among the 165 rare variants in high evidence DCM-related genes, 27 (16.4%) and 59 (35.8%) were interpreted as pathogenic (P) and likely pathogenic (LP), respectively. In addition, 41 (47.7%) and 16 (18.6%) of these 86 P/LP variants are located in *TTN* and *FLNC*, respectively. The *FLNC* group contained more patients with NYHA class IV than the P/LP-negative group (*FLNC*, 16/18 vs. P/LP-negative, 81/123, P = 0.049).

**Conclusions:**

Based on WES, we provided a primary genetic spectrum of DCM patients undergoing HTx in the Chinese population. *TTN* and *FLNC* harbour the most P/LP variants. *FLNC* truncation may lead to severe clinical symptoms in DCM patients.

**Supplementary Information:**

The online version contains supplementary material available at 10.1186/s12967-023-04282-5.

## Background

Dilated cardiomyopathy (DCM) is a myocardial disorder that is characterized by the presence of left ventricular dilatation and systolic impairment in the absence of abnormal loading conditions and severe coronary artery disease [1]. DCM has a prevalence of approximately 36.5 in 100,000 in Western populations and 19 in 100,000 in the Chinese population [2, 3]. Genetic causes account for 30–50% of DCM cases [4, 5]. Titin (*TTN*), lamin A/C (*LMNA*) and myosin heavy chain 7 (*MYH7*) are the most commonly mutated genes associated with DCM; the frequencies of mutations in these genes are 12–25%, 4–8% and 3–4%, respectively [6, 7]. DCM is one of the most common causes of heart failure and heart transplantation (HTx) worldwide [8]. The genetic basis of DCM among patients undergoing HTx, especially those in the Chinese population, remains elusive.

As a next-generation sequencing technology, whole-exome sequencing (WES) has advanced the understanding of genetic nonsyndromic cardiomyopathy over the last decade. WES, in which the protein-coding regions of ~ 25,000 genes are sequenced, has been used to identify 24 putative new disease genes for genetic cardiomyopathies [9]. It has been increasingly used in the diagnostic evaluation of patients with suspected genetic disorders [10]. We believe that WES is an effective and convenient tool for understanding the genetic background and pathogenesis of DCM.

Based on this, we conducted a single-centre retrospective study in which WES was performed for 208 DCM patients (Fuwai DCM HTx cohort) recruited from Fuwai Hospital who underwent HTx due to end-stage heart failure. Our results provide a primary genetic basis for DCM patients undergoing HTx in the Chinese population and will be helpful for DCM molecular diagnosis, progression prediction and clinical therapy.

## Methods

### Patient enrolment

A total of 208 unrelated DCM patients who underwent heart transplantation (HTx) at Fuwai Hospital (Fuwai DCM HTx cohort) from June 2004 to June 2017 were selected. All participants were diagnosed according to the following clinical criteria: (1) left ventricular end-diastolic dimension (LVEDD) > 117% of the predicted value corrected for body surface area and age; (2) left ventricular ejection fraction (LVEF) < 45% in the absence of abnormal loading conditions (hypertension, primary valve disease) or coronary artery disease sufficient to cause global systolic impairment; and (3) hypertension and primary valve disease were excluded by medical history or by cardiac magnetic resonance imaging and echocardiography, and patients with coronary artery disease with stenosis > 50% of at least one main vessel were excluded by coronary angiography relying on experienced clinicians. Familial DCM was defined as at least one additional family member with DCM or in the presence of one relative with sudden cardiac death before 35 years of age [11].

A group of 187 obese children without cardiac defects and 381 patients with transposition of the great arteries was used as the reference, which was reported in an article published previously [12].

### DNA extraction and whole-exome sequencing

WES was performed at Novogene Bioinformatics Technology Co., Ltd. (Beijing, China). Genomic DNA was extracted from transplanted heart tissues using a Magnetic Universal Genomic DNA kit (TIANGEN Biotech, Beijing, China) according to the manufacturer’s protocol. DNA samples with an optical density 260/280 ratio ranging from 1.8 to 2.0 and a content above 1.0 μg were used for library preparation. The Agilent SureSelect Human All Exon V5 kit (Agilent, Santa Clara, CA, USA) was used to capture the exome regions according to the manufacturer’s protocol. First, qualified genomic DNA was randomly fragmented to the 180–280 bp size range by Covaris LE220R-plus (Covaris, USA). Second, DNA fragments were end repaired and phosphorylated, followed by A-tailing and ligation at the 3’ ends with paired-end adapters. PCR was conducted to selectively enrich DNA fragments with ligated adapter molecules on both ends. After PCR, libraries were hybridized in liquid phase with a biotin-labelled probe, and then magnetic beads with streptomycin were used to capture the target exons of genes. Index tags were added through PCR with captured libraries. PCR products were purified using the AMPure XP system (Beckman Coulter, Beverly, USA) and analysed for size distribution by the Agilent 5400 system (AATI) (Agilent, USA). The qualified libraries were sequenced on an Illumina HiSeq X-ten platform (Illumina Inc., San Diego, CA, USA) with the PE150 strategy.

### Raw data quality control and mapping

The FASTQ format files, which contain sequence information and corresponding sequencing quality information, were obtained from the sequencing platform. For the raw data, the percentage of bases with a Phred score greater than 20 to the total bases (Q20) was required to be above 90%. The percentage of bases with a Phred score greater than 30 to the total bases (Q30) was required to be above 80%, and the average error rate of all bases was required to be below 0.1% (Additional file 3: Table S1). The average amount, number of reads and depth of raw data were 12.59 G, 83,943,430, and 250x, respectively (Additional file 3: Table S1). Adapter trimming and quality filtering of the raw data were performed using Trimmomatic v0.93 [13]. The average clean read number was 82,437,280 (Additional file 3: Table S1). The clean data were mapped to the human reference genome (UCSC hg19) using BWA MEM (0.7.17-r1188) under default settings [14]. The average mapped reads were 82,271,411, and the average mapped rate was 99.80% (Table S1). Duplicate reads were marked using MarkDuplicates tools in the Picard toolkit (2.21.1, http://broadinstitute.github.io/picard/). Base quality recalibration was performed using the BaseRecalibrator tool in the Genome Analysis Toolkit (GATK, v4.1.4.0) [15]. The average sequencing depth on target was 145x (Additional file 3: Table S1).

### Variant discovery and quality filtering

Variant discovery and quality filtering followed the GATK best practice pipeline for germline SNPs and indels. In brief, variant calling was performed on samples of cohorts using the HaplotypeCaller algorithm in Genomic Variant Call Format mode, and only the variants located within the capture regions of the Agilent SureSelect Human All Exon V5 kit were retained. Next, variants called from the VCF files of all samples, including DCM and reference samples, were subjected to joint genotyping analysis. Finally, variant quality filtering was performed based on variant quality score recalibration. After completing these processes, 1,507,591 variants were obtained in 208 DCM patients and the reference. The number of variants carried by each individual in DCM patients is shown in Additional file 3: Table S2. The VCF files of 208 DCM patients were deposited in the Genome Variation Map (GVM) [16] in the National Genomics Data Center, Beijing Institute of Genomics, Chinese Academy of Sciences and China National Center for Bioinformation [17], under accession number GVM000540.

### Population structure examination and sample anomaly check

To examine the population structure of the DCM cases and references, we performed principal component analysis (PCA) based on the genotypes of the called variants in all samples. We visualized the first three components in two-dimensional space. Using PLINK [18], we checked the relatedness of all samples. Specifically, Yoruba in Ibadan, Nigeria, Utah residents with Northern and Western European ancestry, Chinese Dai in Xishuangbanna, China, Han Chinese in Beijing, China, and Southern Han Chinese from the HapMap phase3 dataset were used. As shown in Additional file 1: Fig. S1, we first retained variants with two alleles, a call rate greater than 90%, and a minor allele frequency (MAF) greater than 0.05 in all samples and the HapMap phase3 dataset. Then, the 180,463 overlapping variants were pruned using PLINK, considering window sizes of 100 variants, a step size of 25 and a pairwise r^2^ threshold of 0.05 (–indep-pairwise 100 25 0.05), and a total of 15,286 independent SNPs for PCA were retained. The identity-by-decent was calculated with PLINK within the case and reference groups, and no sample was excluded.

### Functional and population frequency annotation of variants

ANNOVAR [19] was used to annotate the variants. Variants predicted to alter the coding sequence of the gene product were classified as truncating or nontruncating variants. Truncating variants include those resulting in frameshifts, premature stop-gain or canonical splice sites, and nontruncating variants include damage missense variants (missense variants with Rare Exome Variant Ensemble Learner > 0.5) [20], stop-loss, and nonframeshift variants. To filter for rare variants, we used allele frequencies of all population exome data documented in a public database: the exome dataset in the Genome Aggregation Database. A variant was considered rare if the allele frequency in this public database was lower than 0.0001 or not documented.

### Gene burden analysis

We followed the steps shown in Additional file 2: Fig. S2 to filter the obtained 1,507,591 variants in 8226 protein-coding genes for gene burden analysis. The number of variants before and after filtering is shown in Additional file 3: Table S2. We ran burden tests with four models, including the CMC, Fp, SkatO, and Zeggini models implemented in the toolkit RVTESTS (v20171009): variable-threshold burden tests with MAF cut-offs of ≤ 5%. The top 3 principal components and sex were used as covariates. We used a significance threshold of P ≤ 6.1 × 10^–6^, corresponding to a Bonferroni correction for 8226 protein-coding genes. A quantile‒quantile plot was generated by the R package qqman [21].

### Variant classification

All variants of the DCM high evidence genes were classified into five categories, including pathogenic (P), likely pathogenic (LP), variant of unknown significance (VUS), likely benign (LB), or benign (B), based on the American College of Medical Genetics and Genomics and the Association for Molecular Pathology (ACMG/AMP) 2015 guidelines.

### Statistical analysis

Continuous variables are expressed as the mean ± SD values, and all categorical variables are depicted using relative frequency distributions. Differences between means were compared using Student’s t test. Comparisons of categorical variables between different groups, such as patients with or without P/LP, were performed using a χ^2^ test where appropriate; otherwise, continuity correction or Fisher’s exact test was used. The statistical analysis was performed in SPSS 20.0 (IBM, USA) or R (https://www.r-project.org/), and a two-sided P value < 0.05 indicated statistical significance.

## Results

### Clinical characteristics of patients enrolled in the Fuwai DCM HTx cohort

The Fuwai DCM HTx cohort comprised 208 unrelated patients with DCM who had undergone heart transplantation. The clinical characteristics are shown in Table 1. One hundred sixty-seven (80.3%) patients were male, and 41 (19.7%) patients were female. All individuals had end-stage heart failure in New York Heart Association (NYHA) classes III–IV. The mean age of onset and heart transplantation was 36.5 ± 11.8 years and 42.4 ± 13.2 years, respectively, and the mean disease course between the diagnosis of DCM and heart transplantation was 71.6 ± 65.6 months. A family history of DCM was identified in 18 (8.7%) individuals by follow-up investigation. The mean value of LVEDD was 75.7 ± 10.8 mm, which is categorized as a severely abnormal left ventricle dimension, and the mean value of LVEF was 23.9 ± 6.4%, indicating severe left ventricle dysfunction.

**Table 1:** Clinical characteristics of 208 patients in the Fuwai DCM HTx cohort

Characteristics	General (n = 208)	Male (n = 167)	Female (n = 41)
Sex (%)	208 (100)	167 (80.3)	41 (19.7)
Height (cm)	170.2 ± 7.8	172.4 ± 6.6	161.3 ± 5.3
Weight (kg)	63.5 ± 12.0	66.2 ± 11.4	52.7 ± 7.8
BMI (kg/m^2^)	21.8 ± 3.4	22.2 ± 3.4	20.2 ± 2.9
NTpro-BNP (× 10^3^ ng/L)	2.8 ± 2.2	2.8 ± 2.2	3.0 ± 2.0
Age of onset (years old)	36.5 ± 11.8	36.1 ± 11.7	37.8 ± 11.9
Age of transplantation (years old)	42.4 ± 13.2	42.3 ± 13.0	42.9 ± 14.1
Disease course (month)	71.6 ± 65.6	74.2 ± 66.4	61.2 ± 61.7
Family history, n (%)	18 (8.7)	15 (9.0)	3 (7.3)
AF, n (%)	58 (27.9)	49 (29.3)	9 (22.0)
Pacemaker, n (%)	40 (19.2)	36 (21.6)	4 (9.8)
Diabetes, n (%)	14 (6.7)	11 (6.6)	3 (7.3)
Echocardiography			
LAD (mm)	49.7 ± 8.8	51.0 ± 9.0	44.5 ± 5.2
LVEDD (mm)	75.7 ± 10.8	77.5 ± 10.4	68.4 ± 9.1
LVEF (%)	23.9 ± 6.4	23.8 ± 6.5	24.2 ± 6.1
IVS (mm)	8.5 ± 1.4	8.7 ± 1.4	8.0 ± 1.5
RVD (mm)	27.0 ± 5.9	27.4 ± 5.8	25.2 ± 5.8
NYHA scale, n (%)			
NYHA class III	61 (29.3)	48 (28.7)	13 (31.7)
NYHA class IV	147 (70.7)	119 (71.3)	28 (68.3)

Continuous variables are expressed as the mean ± SD values, and all categorical variables are depicted using relative frequency distributions

Disease course: months from symptom onset to heart transplantation

*BMI* body mass index, *AF* atrial fibrillation, *LAD* left atrial diameter, *LVEDD* left ventricular end-diastolic dimension, *LVEF* left ventricular ejection fraction, *IVS* interventricular septum, *RVD* right ventricle diameter, *NYHA* New York Heart Association classification

### Gene burden analysis of rare protein-altering variants

**Fig. 1:**
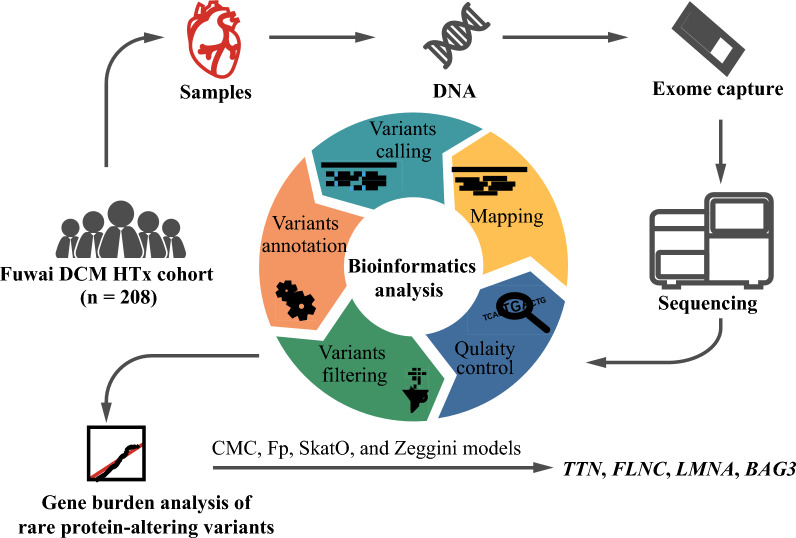
Workflow of whole-exome sequencing and data analysis. Applied workflow, consisting of exome capture, whole-exome sequencing, data processing, and gene burden analysis. DCM, dilated cardiomyopathy; HTx, heart transplantation

**Fig. 2:**
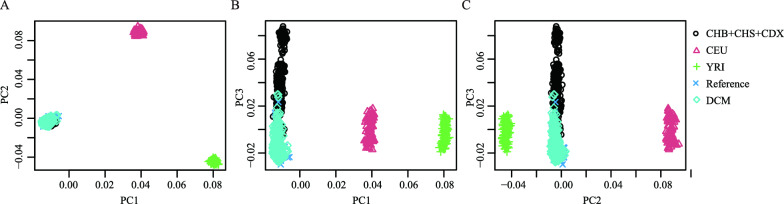
Principal component analysis of the DCM cases, reference group, and East Asian population. Principal component analysis based on common variants confirmed that there were no significant differences in population structure between the DCM cases and reference group. The individual genotypes of human populations are from Hapmap3. **A** PC1 and PC2, **B** PC1 and PC3, **C** PC2 and PC3. *DCM* dilated cardiomyopathy, *YRI* Yoruba in Ibadan, Nigeria, *CEU* Utah Residents with Northern and Western European Ancestry, *CDX* Chinese Dai in Xishuangbanna, China, *CHB* Han Chinese in Beijing, China, *CHS* Southern Han Chinese

The workflow of the study design is shown in Fig. 1. We performed WES for 208 patients to investigate the genetic variants in coding regions of the genome that are associated with DCM. WES data from 187 obese children without heart defects and 381 patients with transposition of the great arteries were used as the reference, which was reported in a previously published article [12]. PCA suggested that the two datasets are comparable in terms of population structure (Fig. 2).

**Fig. 3:**
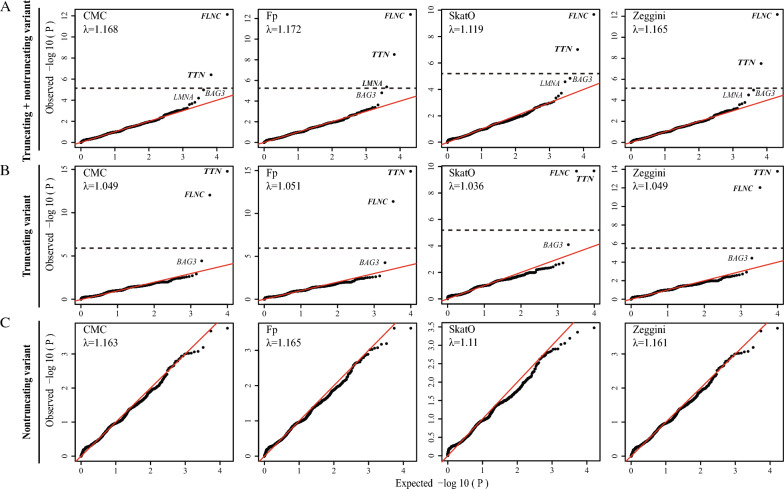
Quantile‒quantile plot of the P values for all associations with four models. Four gene-based tests with a variety of models, including CMC, Fp, SkatO, and Zeggini models implemented in the toolkit RVTESTS (v20171009): variable-threshold burden tests with MAF cut-offs of ≤ 5%. **A** All variants (truncating variants + nontruncating variants) Q–Q plot for DCM cases vs. the reference group. **B** Truncating variant Q–Q plot for DCM cases vs. the reference group. **C** Nontruncating variant Q–Q plot for DCM cases vs. the reference group. *DCM*, dilated cardiomyopathy; *MAF*, minor allele frequency; *Q–Q plot*, quantile‒quantile plot

After variant discovery and quality filtering, we used the deleteriousness prediction and allele frequency in the public database to screen for rare protein-altering variants (see Methods) and obtained 22,622 SNPs and 5545 indels in the exons and canonical splicing regions of 8226 genes for gene burden analysis. Figure 3 shows the quantile‒quantile plot (Q–Q plot) of four different models for gene-burden tests, and Table 2 shows the genes that reached or approached the exome-wide significance level.

**Table 2:** Genes that reached or approached exome-wide significance in the Fuwai DCM HTx cohort

	No. of patients in DCM cases (208)	No. of patients in the reference group (568)	CMC	Fp	SkatO	Zeggini
Truncating + nontruncating						
*TTN*	66	84	**3.90E−07**	**3.06E−09**	**9.66E−08**	**3.17E−08**
*FLNC*	32	12	**7.38E−13**	**4.08E−13**	**2.15E−10**	**6.73E−13**
*LMNA*	9	3	6.35E**−**05	**4.25E−06**	1.44E−05	3.07E−05
*BAG3*	7	0	1.05E−05	1.59E−05	2.64E−05	1.05E−05
Truncating variants						
*TTN*	39	11	**1.66E−15**	**1.22E−15**	**2.15E−10**	**1.64E−14**
*FLNC*	18	0	**9.24E−13**	**3.98E−12**	**2.23E−10**	**9.24E−13**
*LMNA*	4	0	1.21E−03	2.91E−03	5.24E−03	1.21E−03
*BAG3*	6	0	3.71E−05	5.49E−05	8.03E−05	3.71E−05
Nontruncating variants						
*TTN*	38	74	5.95E−02	8.09E−02	1.60E−01	7.79E−02
*FLNC*	16	12	1.76E−04	2.37E−04	3.34E−04	1.76E−04
*LMNA*	5	3	1.06E−02	2.23E−03	7.19E−03	4.44E−03
*BAG3*	1	0	1.22E−01	1.22E−01	1.22E−01	1.22E−01

Burden analysis was performed via four test models (CMC, Fp, SkatO, and Zeggini) in DCM cases and the reference group, and a P value ≤ 6.1 × 10^–6^ (Bonferroni correction for 8226 genes), which is marked in bold, indicated a statistically significant difference

*DCM* dilated cardiomyopathy, *TTN* titin, *FLNC* filamin C, *LMNA* lamin A/C, *BAG3* BAG cochaperone 3

A combined analysis of truncating and nontruncating variants showed that in DCM patients, aggregated protein-altering variants in *TTN* (case, 31.7%, 66/208 vs. reference, 14.8%, 84/568), filamin C (*FLNC*) (case, 15.4%, 32/208 vs. reference, 2.1%, 12/568), and *LMNA* (case, 4.3%, 9/208 vs. reference, 0.5%, 3/568) were significantly enriched (Fig. 3A, Table 2). Protein-truncating variants in *TTN* and *FLNC* were significantly enriched in DCM patients (*TTN*: case, 18.8%, 39/208 vs. reference, 1.9%, 11/568; *FLNC*: case, 8.7%, 18/208 vs. reference, 0%, 0/568) (Fig. 3B, Table 2). For nontruncating variants, we did not identify any genes that reached exome-wide significance (Fig. 3C, Table 2). In addition, BAG cochaperone 3 (*BAG3*), a DCM-associated gene, also showed relatively strong associations with truncating variants through the combined analysis, but this has not yet reached exome-wide significance (Fig. 3A, B).

### Variant classification

Burden analysis of WES strongly suggested that *TTN*, *FLNC*, *LMNA*, and *BAG3* harbour pathogenic rare variants but do not directly inform the interpretation of any single variant in our cohort. To interpret the pathogenicity of a single variant, we conducted an online (http://wintervar.wglab.org/) pathogenicity assessment of these rare variants according to ACMG/AMP 2015 guidelines. We focused on the rare variants among the high evidence genes (*ACTC1*, *ACTN2*, *BAG3*, *DES*, *DSP*, *FLNC*, *JPH2*, *LMNA*, *MYH7*, *NEXN*, *PLN*, *RBM20*, *SCN5A*, *TNNC1*, *TNNI3*, *TNNT2*, *TPM1*, *TTN*, and *VCL*) summarized in the previous review [22]. Among these 19 genes, 16 genes with 165 rare variants were identified in our cohort (Additional file 3: Table S3); these variants included 87 missense, 30 stop-gain, 23 frameshift deletion, 12 frameshift insertion, 7 nonframeshift deletion, and 6 splicing variants. Among these 165 rare variants, 81 have been reported in the Clinvar database (https://www.ncbi.nlm.nih.gov/clinvar/), 37 have been reported in the published papers, and only 4 have been reported in papers which focused on the HTx patients (Additional file 3: Table S3).

According to the ACMG/AMP 2015 guidelines, we found that 27 (16.4%), 59 (35.8%), 73 (44.2%), and 6 (3.6%) variants were interpreted as P, LP, VUS, and LB, respectively (Additional file 3: Table S3). Among these 86 P/LP variants, 41 (47.7%), 16 (18.6%), 8 (9.3%), and 4 (4.7%) were in *TTN*, *FLNC*, *LMNA*, and *BAG3*, respectively. In addition, 32 of these 86 P/LP variants have been reported in the Clinvar database, of which 18 variants are interpreted as P/LP (Table 3).

**Table 3:** Variants interpreted as P/LP by ACMG/AMP 2015 guidelines and in the Clinvar database

Gene	Transcript	Coding change	Protein change	Pathogenicity assessment	Interpretation in Clinvar
*BAG3*	NM_004281	c.C268T	p.R90X	P	P
*DSP*	NM_004415	c.C6496T	p.R2166X	P	P/LP
*FLNC*	NM_001458	c.5842 + 1G > A		P	LP
*FLNC*	NM_001458	c.C805T	p.R269X	P	P
*FLNC*	NM_001458	c.G1730A	p.W577X	P	P
*LMNA*	NM_005572	c.C568T	p.R190W	LP	P
*LMNA*	NM_005572	c.C673T	p.R225X	P	P
*LMNA*	NM_005572	c.G4T	p.E2X	P	P
*MYH7*	NM_000257	c.C427T	p.R143W	LP	P/LP
*MYH7*	NM_000257	c.G1106A	p.R369Q	LP	LP
*TNNT2*	NM_000364	c.650_652del	p.217_218del	LP	P/LP
*TNNT2*	NM_000364	c.C418T	p.R140C	P	P/LP
*TNNT2*	NM_000364	c.C451T	p.R151W	P	P
*TTN*	NM_001256850	c.82741_82748del	p.D27581fs	P	LP
*TTN*	NM_001256850	c.89421_89424del	p.K29807fs	P	P/LP
*TTN*	NM_001256850	c.C80344T	p.R26782X	P	LP
*TTN*	NM_001256850	c.C82117T	p.R27373X	P	P
*TTN*	NM_001256850	c.C95419T	p.R31807X	P	LP

*ACMG/AMP* American College of Medical Genetics and Genomics and the Association for Molecular Pathology, *BAG3* BAG cochaperone 3, *DSP* desmoplakin, *FLNC* filamin C, *LMNA* lamin A/C, LP likely pathogenic, *MYH7* myosin heavy chain 7, *P* pathogenic, *TNNT2* troponin T2, cardiac type, *TTN* titin

### Genotype in relation to clinical characteristics

We first compared clinical characteristics between P/LP-positive and P/LP-negative patients, and no significant difference was observed (Additional file 3: Table S4). Then, we compared clinical characteristics between the P/LP-negative group and different variant groups (*TTN*, *FLNC*, *LMNA*, and *BAG3* groups) (Additional file 3: Table S4). Compared with the P/LP-negative group, the *TTN* group exhibited a longer left atrial diameter (LAD) (*TTN*, 52.1 ± 9.2 mm vs. P/LP-negative, 48.9 ± 8.7 mm, P = 0.048) (Additional file 3: Table S4). The *FLNC* group contained more patients with NYHA class IV than the P/LP-negative group (*FLNC*, 16/18 vs. P/LP-negative, 81/123, P = 0.049) (Additional file 3: Table S4). The *LMNA* group exhibited more frequent pacemaker implantation (*LMNA*, 5/8 vs. P/LP-negative, 26/123, P = 0.025) and smaller LVEDD (*LMNA*, 65.0 ± 6.6 mm vs. P/LP-negative, 76.7 ± 11.3 mm, P = 0.005) than the P/LP-negative group (Additional file 3: Table S4). In addition, we compared the clinical characteristics of individuals carrying one P/LP variant with those carrying more than one P/LP variant, and no significant differences were observed (Additional file 3: Table S5).

## Discussion

In the present study, we performed WES-based genetic screening for 208 unrelated DCM patients undergoing HTx in the Chinese population. *TTN*, *FLNC* and *LMNA* were the main genetic causes, in which rare protein-altering variants were significantly enriched. Among the 165 rare variants in DCM high evidence genes, 86 were interpreted as P/LP. *TTN* and *FLNC* harboured the most P/LP variants, as they harboured 41 (47.7%) and 16 (18.6%), respectively.

As the largest known protein, TTN spans half of the cardiac sarcomere, which is the basic structural and functional unit of striated muscle. It is essential for heart development as well as the mechanical and regulatory function of sarcomeres [23]. The most common genetic predisposition to DCM is truncating variants in *TTN*, which occur in up to 15% of all DCM patients and up to 25% of severe, end-stage, or familial DCM cases [24]. In line with a previous study that focused on the genetic risk of early-onset sporadic DCM in the Chinese Han population [25], this study found that *TTN* truncations were the most common truncating variant, and they existed in 18.8% (39/208) of DCM cases in our cohort. In addition, we found that the *TTN* group exhibited a larger LAD than the P/LP-negative group. This finding has not yet been reported, and larger sample size studies are needed to verify this.

*FLNC* is specifically expressed in striated muscle. It acts as an actin-crossing linker to organize actin filaments, which play a vital role in the structural integrity and cell signalling of the sarcomere [26]. *FLNC* variants have been shown to play a vital role in the pathogenesis of cardiomyopathies [27, 28]. Nontruncated *FLNC* tends to result in hypertrophic cardiomyopathy and restrictive cardiomyopathy, and truncated *FLNC* tends to result in DCM and arrhythmogenic right ventricular cardiomyopathy [29, 30]. In our cohort, 8.7% (18/208) of patients carried *FLNC* truncating variants, and this frequency was much higher than that in European and North American DCM cohorts (1%) [7]. This may be caused by ethnic differences in the genetic background. However, in a previous study in a Chinese population [25], only three of 363 DCM patients carried *FLNC* truncating variants, which is much less than our cohort. Another possible explanation is that *FLNC* truncations may lead to severe heart failure; this requires more significant interventions, such as left ventricular assist devices or HTx. This hypothesis is partly supported by our statistical test results that the *FLNC* group contained more patients with NYHA class IV than the P/LP-negative group.

The *LMNA* gene mainly encodes lamin A and lamin C, which are the main constituents of the nuclear lamina underneath the inner nuclear membrane. *LMNA* mutations can lead to a group of progeroid laminopathies, including cardiovascular disorders [31]. The *LMNA* variants carried by DCM patients are inherited in an autosomal dominant pattern, which is characterized by abnormal conduction and malignant ventricular arrhythmia [32]. This may explain the higher frequency of pacemaker implantation in the *LMNA* group than in the P/LP-negative group in our cohort. Reportedly, minor systolic dysfunction without ventricular dilatation could be observed in some *LMNA* mutation carriers [33], which is consistent with the significantly lower LVEDD in the *LMNA* group than in the P/LP-negative group. Since all patients included in this study were required to meet the inclusion criteria “LVEDD > 117% of the predicted value corrected for body surface area and age”, it is possible that some *LMNA* mutation carriers were excluded from this study.

Due to the lack of sufficient case evidence and experimental evidence, many variants in DCM genes are classified as VUS [22]. Our research has also encountered this situation, especially for many missense variants. For the frameshift and splicing variants in high evidence genes, although some adjudication criteria cannot be well applied, the majority of them are evaluated as LP according to the criteria PVS1 and PM2. Since variant classification is a dynamic and probabilistic process that can change over time [34, 35], we consider that the existing pathogenicity classification of these variants is not conclusive. With the increase in clinical evidence and experimental research on specific variants in the future, we believe that the pathogenicity of variants that were currently classified as VUS in the Fuwai DCM HTx cohort will be more clearly interpreted.

This study has several limitations. First, utilizing published data as a reference population dataset for making comparisons with DCM patients presents inherent limitations. These limitations include the unknown prevalence of DCM among individuals in the reference group, the utilization of published data to define rare variants and as a control cohort, and the capture region difference between different exon capture kits (the Agilent SureSelect Human All Exon V5 kit in our study and the NEBNext® Ultra DNA Library Prep Kit (Illumina Inc., San Diego, CA, USA) or NimbleGen’s SeqCap EZ Human Exome Library v3.0 Kit (Roche, Pleasanton, CA, USA) in the study by Liu et al.) [12], which may introduce biases. However, our sequencing quality control based on coverage region, depth of variants (with a minimum depth of 10 for more than 90% individuals in both groups), and adjustment for population structure (including sex and the first three PCA as covariates) allowed for the calibration of burden testing results. The results of the gene burden analysis also showed that the inflation factor lambda was close to 1, indicating that the system error was small and that the statistical results were relatively reliable.

Second, survival bias cannot be disregarded, which may result in cohorts being depleted of variants that cause severe early-onset DCM. In addition, some individuals in the reference group may develop DCM in the future. Given the relatively low prevalence of DCM (19 in 100,000 in the Chinese population) [3], which could result in less than one individual in the reference group potentially developing DCM in the future, we infer that this bias will not significantly impact our findings.

Third, our investigation centred on variants within protein-coding regions, as the employed technology did not comprehensively identify other variant categories, including noncoding, epigenetic, and large structural variants. Subsequent research based on whole-genome sequencing of individuals with DCM will explore these matters.

Finally, the number of patients enrolled was relatively low, and no replication cohort was provided in our study. Future studies among larger cohorts and replication cohorts will be crucial to further confirm our findings. Over 80% of DCM patients in our cohort were male, and more genetic characteristics of female DCM patients could be investigated in the future.

## Conclusions

Here, we provided a primary genetic mutation spectrum of DCM patients undergoing HTx based on WES in the Chinese population, which could lay a foundation for the molecular diagnosis, progression prediction and clinical therapy of DCM patients. *TTN* and *FLNC* harbour the most P/LP variants. *FLNC* truncation may lead to severe clinical symptoms in DCM patients.

## Supplementary Information

**Additional file 1: Figure S1.** Variant filtering flowchart for PCA. DCM, dilated cardiomyopathy; MAF, minor allele frequency; PCA, principal component analysis.

**Additional file 2: Figure S2.** Variant filtering flowchart for gene burden analysis. DCM, dilated cardiomyopathy; MAF, minor allele frequency; REVEL, rare exome variant ensemble learner.

**Additional file 3: Table S1.** Quality control statistics for sequencing. DCM, dilated cardiomyopathy; Q20, percentage of bases with a Phred score greater than 20 to the total bases; Q30, percentage of bases with a Phred score greater than 30 to the total bases. **Table S2.** The number of variants carried by each DCM patient before and after filtering. DCM, dilated cardiomyopathy. **Table S3.** Rare variants in high evidence genes identified in the Fuwai DCM HTx cohort. One *TTN* variant (NM_133379:exon46:c.C12949T:p.Q4317X) was predicted to be VUS since it affects only the Novex-3 isoform. Bold PMID represents the paper focused on the HTx patients. ACMG/AMP, American College of Medical Genetics and Genomics and the Association for Molecular Pathology; *ACTC1*, actin alpha cardiac muscle 1; *ACTN2*, actinin alpha 2; B, benign; *BAG3*, BAG cochaperone 3; DCM, dilated cardiomyopathy; *DES*, desmin; *DSP*, desmoplakin; *FLNC*, filamin C; HTx, heart transplantation; *JPH2*, junctophilin 2; LB, likely benign; *LMNA*, lamin A/C; LP, likely pathogenic; *MYH7*, myosin heavy chain 7; *NEXN*, nexilin F-actin binding protein; P, pathogenic; *PLN*, phospholamban; *RBM20*, RNA binding motif protein 20; *SCN5A*, sodium voltage-gated channel alpha subunit 5; *TNNT2*, troponin T2, cardiac type; *TTN*, titin; VUS, variant of unknown significance. **Table S4.** The clinical characterization of DCM in relation to P/LP variant status. Continuous variables are expressed as the mean ± SD values, and all categorical variables are depicted using relative frequency distributions. Disease course: months from symptom onset to heart transplantation. AF, atrial fibrillation; *BAG3*, BAG cochaperone 3; BMI, body mass index; DCM, dilated cardiomyopathy; *FLNC*, filamin C; IVS, interventricular septum; LAD, left atrial diameter; *LMNA*, lamin A/C; LP, likely pathogenic; LVEDD, left ventricular end-diastolic dimension; LVEF, left ventricular ejection fraction; NYHA, New York Heart Association classification; P, pathogenic; RVD, right ventricle diameter; *TTN*, titin. P value < 0.05 = *, < 0.01 = **. **Table S5.** Clinical characterization of DCM in patients with one P/LP variant and those with more than one P/LP variant. Continuous variables are expressed as the mean ± SD values, and all categorical variables are depicted using relative frequency distributions. Disease course: months from symptom onset to heart transplantation. AF, atrial fibrillation; BMI, body mass index; DCM, dilated cardiomyopathy; IVS, interventricular septum; LAD, left atrial diameter; LP, likely pathogenic; LVEDD, left ventricular end-diastolic dimension; LVEF, left ventricular ejection fraction; NYHA, New York Heart Association classification; P, pathogenic; RVD, right ventricle diameter.

## Data Availability

The VCF files of DCM patients were deposited in the Genome Variation Map (GVM) in the National Genomics Data Center, Beijing Institute of Genomics, Chinese Academy of Sciences and China National Center for Bioinformation, under accession number GVM000540. The datasets used and/or analysed during the current study are available from the corresponding author upon reasonable request.

## Abbreviations

ACMG/AMP: American College of Medical Genetics and Genomics and the Association for Molecular Pathology
*ACTC1*: Actin alpha cardiac muscle 1
*ACTN2*: Actinin alpha 2
AF: Atrial fibrillation
B: Benign
*BAG3*: BAG cochaperone 3
BMI: Body mass index
CDX: Chinese Dai in Xishuangbanna, China
CEU: Utah Residents with Northern and Western European Ancestry
CHB: Han Chinese in Beijing, China
CHS: Southern Han Chinese
DCM: Dilated cardiomyopathy
*DES*: Desmin
*DSP*: Desmoplakin
*FLNC*: Filamin C
HTx: Heart transplantation
*JPH2*: Junctophilin 2
LAD: Left atrial diameter
LB: Likely benign
*LMNA*: Lamin A/C
LP: Likely pathogenic
LVEDD: Left ventricular end-diastolic dimension
LVEF: Left ventricular ejection fraction
MAF: Minor allele frequency
*MYH7*: Myosin heavy chain 7
*NEXN*: Nexilin F-actin binding protein
NYHA: New York Heart Association
P: Pathogenic
PCA: Principal component analysis
*PLN*: Phospholamban
Q20: Percentage of bases with a Phred score greater than 20 to the total bases
Q30: Percentage of bases with a Phred score greater than 30 to the total bases
Q–Q plot: Quantile‒quantile plot
*RBM20*: RNA binding motif protein 20
REVEL: Rare exome variant ensemble learner
RVD: Right ventricle diameter
*SCN5A*: Sodium voltage-gated channel alpha subunit 5
*TNNC1*: Troponin C1, slow skeletal and cardiac type
*TNNT2*: Troponin T2, cardiac type
*TPM1*: Tropomyosin 1
*TTN*: Titin
VUS: Variant of unknown significance
*VCL*: Vinculin
WES: Whole-exome sequencing
YRI: Yoruba in Ibadan, Nigeria

## References

[1] Elliott P, Andersson B, Arbustini E (2008). Classification of the cardiomyopathies: a position statement from the European Society of Cardiology Working Group on Myocardial and Pericardial Diseases. Eur Heart J.

[2] McNally EM, Barefield DY, Puckelwartz MJ (2015). The genetic landscape of cardiomyopathy and its role in heart failure. Cell Metab.

[3] Pan Y (1992). A population-based study on incidence of idiopathic cardiomyopathy in Nanjing, 1985–1989. Zhonghua Liu Xing Bing Xue Za Zhi.

[4] Akinrinade O, Ollila L, Vattulainen S (2015). Genetics and genotype-phenotype correlations in Finnish patients with dilated cardiomyopathy. Eur Heart J.

[5] Haas J, Frese KS, Peil B (2015). Atlas of the clinical genetics of human dilated cardiomyopathy. Eur Heart J.

[6] Hershberger RE, Hedges DJ, Morales A (2013). Dilated cardiomyopathy: the complexity of a diverse genetic architecture. Nat Rev Cardiol.

[7] McNally EM, Mestroni L (2017). Dilated cardiomyopathy: genetic determinants and mechanisms. Circ Res.

[8] Lakdawala NK, Winterfield JR, Funke BH (2013). Dilated cardiomyopathy. Circ Arrhythm Electrophysiol.

[9] Spracklen TF, Keavney B, Laing N (2022). Modern genomic techniques in the identification of genetic causes of cardiomyopathy. Heart.

[10] Yang Y, Muzny DM, Xia F (2014). Molecular findings among patients referred for clinical whole-exome sequencing. JAMA.

[11] Mestroni L, Maisch B, McKenna WJ (1999). Guidelines for the study of familial dilated cardiomyopathies. Collaborative Research Group of the European Human and Capital Mobility Project on Familial Dilated Cardiomyopathy. Eur Heart J..

[12] Liu X, Chen W, Li W (2020). Exome-based case-control analysis highlights the pathogenic role of ciliary genes in transposition of the great arteries. Circ Res.

[13] Bolger AM, Lohse M, Usadel B (2014). Trimmomatic: a flexible trimmer for Illumina sequence data. Bioinformatics.

[14] Li H. Aligning sequence reads, clone sequences and assembly contigs with BWA-MEM. Published online May 26, 2013. http://arxiv.org/abs/1303.3997.

[15] McKenna A, Hanna M, Banks E (2010). The Genome Analysis Toolkit: a MapReduce framework for analyzing next-generation DNA sequencing data. Genome Res.

[16] Li C, Tian D, Tang B (2021). Genome Variation Map: a worldwide collection of genome variations across multiple species. Nucleic Acids Res.

[17] CNCB-NGDC Members and Partners (2022). Database Resources of the National Genomics Data Center, China National Center for Bioinformation in 2022. Nucleic Acids Res.

[18] Purcell S, Neale B, Todd-Brown K (2007). PLINK: a tool set for whole-genome association and population-based linkage analyses. Am J Hum Genet.

[19] Wang K, Li M, Hakonarson H (2010). ANNOVAR: functional annotation of genetic variants from high-throughput sequencing data. Nucleic Acids Res.

[20] Ioannidis NM, Rothstein JH, Pejaver V (2016). REVEL: an ensemble method for predicting the pathogenicity of rare missense variants. Am J Hum Genet.

[21] Turner, S.D. qqman: an R package for visualizing GWAS results using Q–Q and manhattan plots. BioRxiv. 2017.

[22] Hershberger RE, Cowan J, Jordan E (2021). The complex and diverse genetic architecture of dilated cardiomyopathy. Circ Res.

[23] Tabish AM, Azzimato V, Alexiadis A (2017). Genetic epidemiology of titin-truncating variants in the etiology of dilated cardiomyopathy. Biophys Rev.

[24] Ware JS, Cook SA (2018). Role of titin in cardiomyopathy: from DNA variants to patient stratification. Nat Rev Cardiol.

[25] Xiao L, Wu D, Sun Y (2022). Whole-exome sequencing reveals genetic risks of early-onset sporadic dilated cardiomyopathy in the Chinese Han population. Sci China Life Sci.

[26] Verdonschot JAJ, Vanhoutte EK, Claes GRF (2020). A mutation update for the FLNC gene in myopathies and cardiomyopathies. Hum Mutat.

[27] Begay RL, Tharp CA, Martin A (2016). FLNC gene splice mutations cause dilated cardiomyopathy. JACC Basic Transl Sci.

[28] Brodehl A, Ferrier RA, Hamilton SJ (2016). Mutations in FLNC are associated with familial restrictive cardiomyopathy. Hum Mutat.

[29] Dungu JN, Langley SG, Hardy-Wallace A (2022). Dilated cardiomyopathy: the role of genetics, highlighted in a family with Filamin C (FLNC) variant. Heart.

[30] Song S, Shi A, Lian H (2022). Filamin C in cardiomyopathy: from physiological roles to DNA variants. Heart Fail Rev.

[31] Cenni V, Capanni C, Mattioli E (2020). Lamin A involvement in ageing processes. Ageing Res Rev.

[32] Orphanou N, Papatheodorou E, Anastasakis A (2022). Dilated cardiomyopathy in the era of precision medicine: latest concepts and developments. Heart Fail Rev.

[33] Holmström M, Kivistö S, Heliö T (2011). Late gadolinium enhanced cardiovascular magnetic resonance of lamin A/C gene mutation related dilated cardiomyopathy. J Cardiovasc Magn Reson.

[34] Jordan E, Peterson L, Ai T (2021). Evidence-based assessment of genes in dilated cardiomyopathy. Circulation.

[35] Westphal DS, Burkard T, Moscu-Gregor A (2020). Reclassification of genetic variants in children with long QT syndrome. Mol Genet Genomic Med.

