# Supplementary figures and images for: Genetic characterization of dilated cardiomyopathy patients undergoing heart transplantation in the Chinese population by whole-exome sequencing
Source: J Transl Med. 2023 Jul 17;21:476. doi: 10.1186/s12967-023-04282-5 (PMC10351148; doi:10.1186/s12967-023-04282-5)

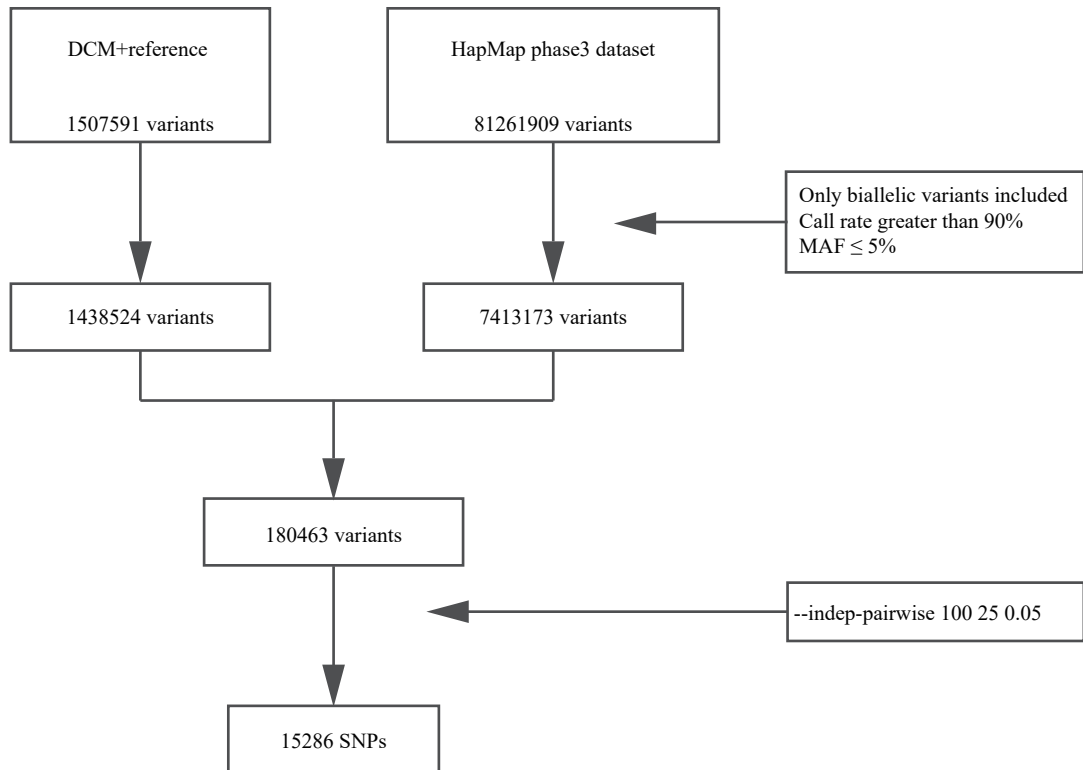

Supplement: Supplementary file 1 — Additional file 1: Figure S1. Variant filtering flowchart for PCA. DCM, dilated cardiomyopathy; MAF, minor allele frequency; PCA, principal component analysis. [file 12967_2023_4282_MOESM1_ESM.pdf]

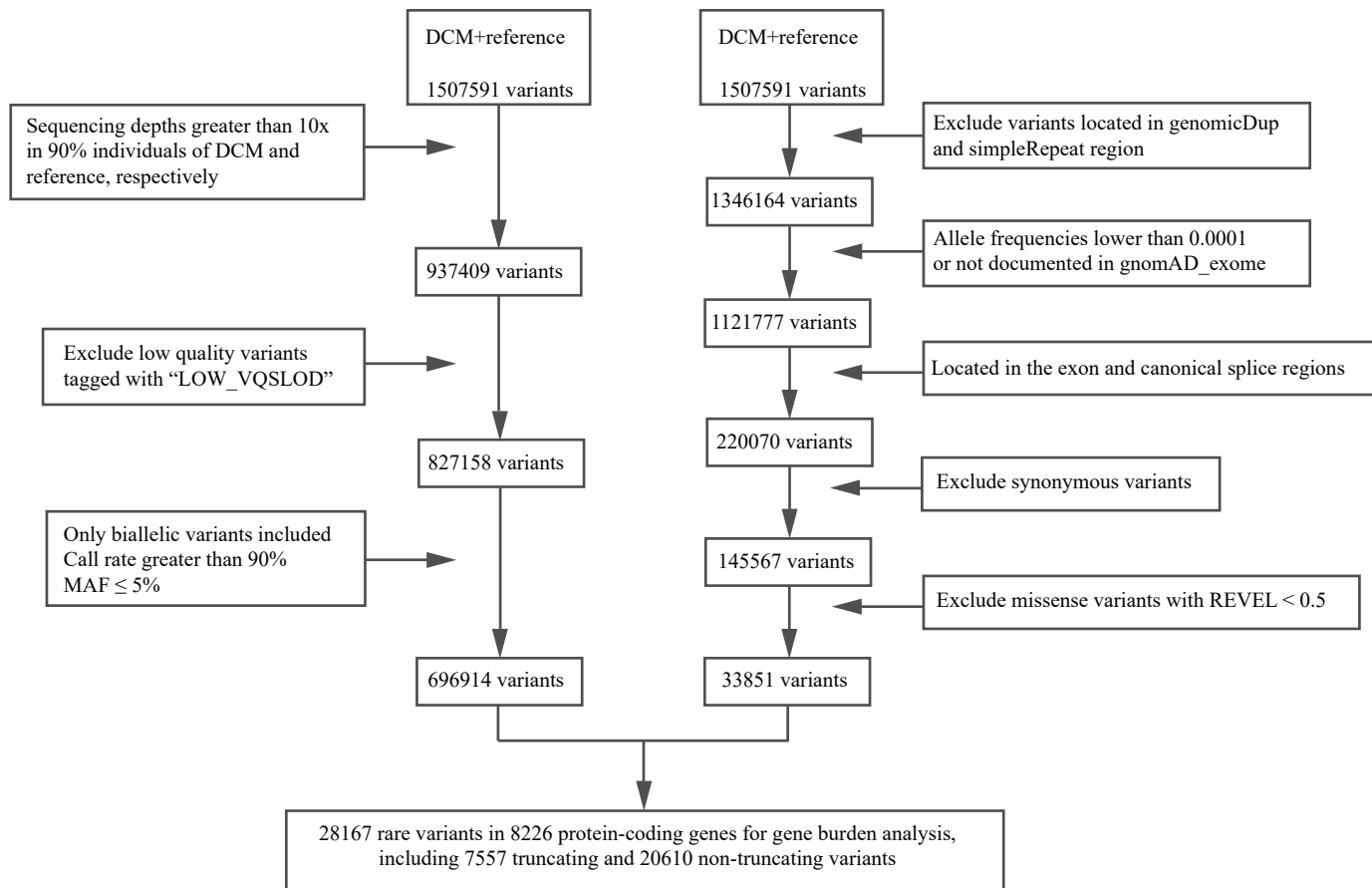

Supplement: Supplementary file 2 — Additional file 2: Figure S2. Variant filtering flowchart for gene burden analysis. DCM, dilated cardiomyopathy; MAF, minor allele frequency; REVEL, rare exome variant ensemble learner. [file 12967_2023_4282_MOESM2_ESM.pdf]
